# Landau–Kleffner syndrome (LKS) in an 8-year-old girl: a case report and review of the literature

**DOI:** 10.1097/MS9.0000000000002156

**Published:** 2024-05-15

**Authors:** Mohammed M. Salahaldin, Mohammad Hakam Shehadeh, Abdullah Abu Keshek, Tala Watheq Abdullah, Hany Abueita

**Affiliations:** a Faculty of Medicine, Al-Quds University, Jerusalem; b Department of Pediatric Neurology, Palestinian Medical Complex (PMC), Ramallah, Palestine

**Keywords:** electroencephalogram, epileptic encephalopathy, Landau–Kleffner syndrome (LKS), language regression, speech therapy

## Abstract

**Introduction and importance:**

Landau–Kleffner syndrome (LKS) is a rare epileptic encephalopathy characterized by language regression and abnormal electroencephalogram (EEG) patterns. This case report highlights the importance of early recognition and intervention in LKS, as well as the challenges in diagnosis and management due to its varied clinical manifestations.

**Case presentation:**

An 8-year-old girl presented with delayed speech, suspected hearing loss, and regression in language skills. Diagnostic tests revealed mild sensorineural hearing loss and EEG abnormalities consistent with LKS. The patient underwent speech therapy and received pharmacological treatment with valproic acid, resulting in significant improvements in language function.

**Clinical discussion:**

This case report provides insights into the typical features of LKS, including language regression and EEG abnormalities. It also highlights uncommon findings such as sensorineural hearing loss and mild intellectual delay. The multidisciplinary approach involving neurology, audiology, speech therapy, and education is crucial in the diagnosis and management of LKS.

**Conclusion:**

Early recognition and intervention, along with tailored pharmacological approaches and a multidisciplinary care approach, are essential in managing LKS. Further research is needed to better understand the pathophysiology, natural history, and optimal treatment of LKS, aiming to improve long-term outcomes for affected children and their families.

## Introduction

Highlights

- Landau–Kleffner syndrome (LKS) is a rare epileptic encephalopathy characterized by language regression and abnormal electroencephalogram (EEG) patterns.
- Diagnosis of LKS is based on clinical features, EEG findings, and exclusion of other causes of language impairment.
- The prognosis of LKS varies depending on the age of onset, duration of symptoms, severity of language impairment, and response to treatment.
- Treatment options for LKS include antiepileptic drugs, corticosteroids, immunoglobulins, speech therapy, and educational interventions.

Landau–Kleffner syndrome (LKS) is a rare epileptic encephalopathy that affects language development and comprehension in children^1,2^. It is characterized by acquired aphasia, which is an inability to understand or produce speech, and abnormal electroencephalogram (EEG) patterns, which show epileptiform activity mainly in the temporoparietal regions of the brain^1,2^. William Landau and Frank Kleffner first reported the syndrome in 1957, based on six cases of children who developed aphasia and seizures^3^. An epidemiologic study revealed that the prevalence of children with LKS in Japan was approximately 1 in a million^4^. Typically, LKS manifests between the ages of 3 and 7 years^5^. Diagnosis of LKS is based on clinical features, EEG findings, and exclusion of other causes of language impairment. The treatment of LKS is challenging and often requires a multidisciplinary approach involving antiepileptic drugs, corticosteroids, immunoglobulins, speech therapy, and educational interventions^6^. The prognosis of LKS varies depending on the age of onset, duration of symptoms, severity of language impairment, and response to treatment. Some children may recover partially or fully, while others may have persistent language deficits and cognitive impairments^6^.

In this case report, we present an 8-year-old girl with LKS. We also review the current literature on LKS and discuss the challenges and controversies in its diagnosis and management. This case report has been reported in line with the CARE criteria^7^.

## Case presentation

A female patient, 8 years old, was referred to the pediatric outpatient department by her mother due to concerns regarding delayed speech and suspected hearing loss. The patient’s mother reported a regression in language skills and was unable to understand or recognize spoken language. The patient was born following an uncomplicated pregnancy and labor, without the need for admission to the neonatal intensive care unit. Additionally, the patient had received all the necessary vaccinations according to the recommended schedule. The patient’s parents were first cousins, and she had five healthy siblings. Importantly, there was no known family history of seizures. The patient had a history of delayed speech since the age of 3 and was undergoing speech therapy. Neurological examination revealed mild intellectual delay, but no motor delay, abnormal movements, aggressive behavior, sleep disturbances, repetitive stereotypical movements, nystagmus, ataxia, tremors, or skin lesions were observed. The patient demonstrated appropriate social interaction, eye contact, knowledge of body parts, toilet training, and self-dressing abilities.

Diagnostic tests were conducted to evaluate the patient’s condition. Auditory Brainstem Response (ABR) and Otoacoustic Emissions (OAE) tests revealed normal results in the right ear, but mild sensorineural hearing loss (SNHL) was detected in the left ear. Tympanometry results were within the normal range for both ears. Brain computed tomography (CT) scans showed no abnormalities, and brain magnetic resonance imaging (MRI) with gadolinium contrast exhibited normal findings. Repeat ABR and OAE tests conducted later revealed normal results bilaterally, and the patient showed mild improvement during speech therapy. However, further evaluation was recommended due to difficulties in reading, learning, and auditory agnosia.

**Figure 1:**
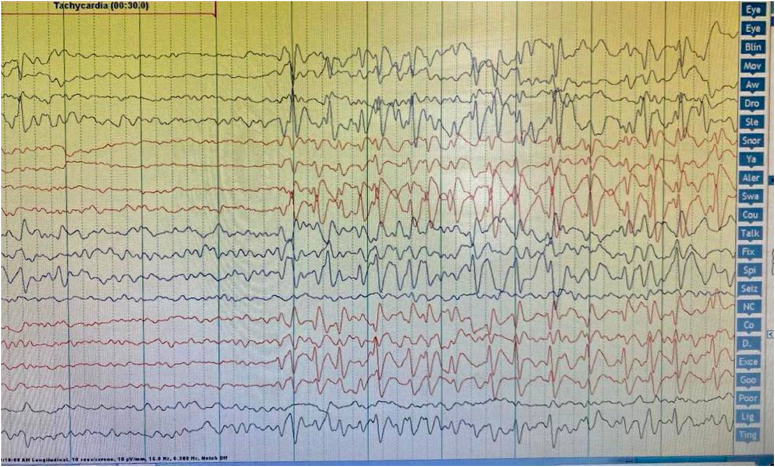
Electroencephalogram in an 8-year-old girl with Landau–Kleffner syndrome. Note the awake background (left) and electrographic status of epilepticus of slow-wave sleep in non-rapid eye movement sleep (right).

The therapeutic interventions employed for the patient included speech therapy, which resulted in mild improvements in speech delay and hearing impairment. Additionally, a referral was made for the patient to attend a special education center to address mild intellectual delay and learning difficulties. The patient’s progress was closely monitored through regular evaluations, follow-up visits, and assessments of language and cognitive abilities. During an EEG conducted, very frequent bilateral fronto-parieto-temporal slow spike-wave discharges with frequent generalized spike-wave activity of 2–3 Hz/s were noted (Fig. 1). The EEG also revealed intermittent focal and generalized epileptic activity associated with verbal agnosia. To rule out seizures during slow-wave sleep, a sleep EEG was recommended, particularly considering the possibility of LKS. The subsequent plan included a referral for a 24-h EEG, initiation of levetiracetam (250 mg twice daily), and ongoing monitoring of the patient’s condition. Upon admission to the hospital, a 24-h EEG revealed electrographic status epilepticus of slow-wave sleep (ESES), which confirmed the diagnosis of LKS. This led to the administration of pulse steroids and tapering prednisone. Throughout the next week, the steroid dosage was gradually reduced. Over the next few months, despite the initial improvements observed in language function following speech therapy and pharmacological treatment with levetiracetam, the patient underwent a follow-up prolonged EEG monitoring, which revealed persistent EEG abnormalities, which suggests that further interventions may be necessary to achieve optimal management, levetiracetam was discontinued and replaced with valproic acid (300 mg twice daily) and the introduction of clobazam (10 mg once daily). After 4 months, a follow-up evaluation was conducted to assess the patient’s neurological, linguistic, and psychosocial development. During this period, the patient continued speech therapy and received ongoing pharmacological treatment with valproic acid. The follow-up visit revealed encouraging results, as the patient demonstrated further improvements in language function and behavior. The EEG abnormalities showed a significant reduction, indicating a positive response to the treatment. The patient’s parents reported that she was more engaged in social interactions and exhibited enhanced cognitive abilities. The patient’s progress was closely monitored, and follow-up visits were scheduled to assess the long-term effects of the interventions and make any necessary adjustments.

## Discussion

In this case report, we presented the clinical features, diagnosis, and treatment of an 8-year-old girl with LKS, a rare syndrome of childhood epilepsy with acquired aphasia. Our case illustrates some of the typical aspects of LKS, as well as some uncommon findings that may expand the current understanding of this syndrome.

The main features of LKS are the onset of epileptic seizures and the progressive loss of language skills in previously normal children, usually between the ages of 3 and 7 years with a higher prevalence among males^3–6^. While the cause and mechanism of LKS are still unclear, some genetic and immune factors have been implicated^4,8,9^. Our patient, who began experiencing symptoms at the age of 3 years, exhibited a progressive decline in both expressive and receptive language abilities over the subsequent years, which aligns with the commonly observed pattern in LKS. The diagnosis of LKS is based on the clinical features, the neuropsychological assessment, EEG findings that are enhanced during sleep, and the exclusion of other causes of acquired aphasia and epilepsy. The hallmark of LKS is the presence of continuous spike-and-wave discharges during slow-wave sleep (CSWS), which are thought to interfere with the maturation of language networks in the brain^10^. However, the diagnosis can be challenging and delayed, as LKS can be misdiagnosed as autism, hearing impairment, learning disability, attention-deficit hyperactivity disorder (ADHD), or other conditions^11^. Our patient showed CSWS in her sleep EEG, as well as focal epileptiform activity in the left temporal region during wakefulness. She also underwent a comprehensive language evaluation, which revealed a severe impairment of both expressive and receptive language functions, consistent with a diagnosis of LKS.

The treatment and prognosis of LKS are variable and depend on several factors, such as the age of onset, the type and frequency of seizures, the degree and type of language impairment, the response to treatment, and the presence of comorbidities. Various pharmacological and non-pharmacological interventions have been proposed for LKS, such as corticosteroids, antiepileptic drugs, vagus nerve stimulation, ketogenic diet, immunoglobulins, or speech therapy^12^. However, the evidence for the efficacy and safety of these treatments is limited and inconsistent, and the optimal timing and duration of treatment are unclear. Moreover, the response to treatment varies among individuals, and some children may not show any improvement or may even worsen despite treatment. The main goals of treatment are to control seizures, improve language function, and prevent cognitive and behavioral deterioration. The prognosis for children with LKS varies as well as is influenced by several factors, such as the age of onset, the type and duration of seizures, the degree and type of language impairment, the response to treatment, and the presence of comorbidities^13^. The prognosis is generally better for children who have an earlier onset, shorter duration, and milder degree of language impairment, as well as a good response to treatment. Some children may recover partially or fully from LKS, while others may have persistent language deficits, cognitive impairment, or behavioral problems. The long-term follow-up of LKS is therefore important to monitor the child’s neurological, linguistic, and psychosocial development and to adjust the treatment accordingly. Our patient was initially treated with levetiracetam, which partially improved her language and behavior. However, due to persistent EEG abnormalities and the need for enhanced control, a switch to valproic acid and introduce clobazam. Clobazam has been documented to significantly reduce continuous spike-wave discharges and has been associated with improvements in language^14^. This transition yielded a significant reduction in EEG abnormalities and a marked improvement in language and behavior. The effectiveness of valproic acid can be attributed to its dual action on GABAergic transmission and ion channels, which are key elements of its complex mechanism of action^15^. By modulating GABAergic transmission and inhibiting sodium and calcium ion channels, valproic acid effectively reduces neuronal hyperexcitability, a characteristic feature of seizures. This mechanism of action has consistently demonstrated its efficacy in seizure management^15^. This suggests that valproic acid may be an effective and well-tolerated option for some patients with LKS.

One of the uncommon findings in our case was the mild sensorineural hearing loss in the left ear, which is not a typical feature of LKS. However, this could be a transient phenomenon or a coincidental finding. The coexistence of LKS with hearing loss and the absence of typical neurological manifestations posed unique challenges in confirming the diagnosis and differentiating LKS from other conditions. Another unusual finding in our case was the presence of a mild intellectual delay, which is not always present in LKS. Some patients may have normal or above-average intelligence, whereas others may have cognitive deficits or behavioral problems. Additionally, the absence of seizures in the patient is noteworthy, as they are reported in ~70% of LKS cases^16^. However, it is important to acknowledge that seizures may manifest later in the disease progression or may be subclinical.

LKS is a rare and complex disorder that poses diagnostic and therapeutic challenges for clinicians. The case we presented illustrates the importance of early recognition and intervention of LKS, as well as the need for a multidisciplinary approach that includes neurology, audiology, speech therapy, and education. We also highlight the possibility of co-occurring conditions such as hearing loss or autism spectrum disorder. Further research is needed to elucidate the pathophysiology, natural history, and optimal treatment of LKS, as well as to improve the quality of life and long-term outcomes of affected children and their families.

## Conclusion

LKS is a rare epileptic encephalopathy characterized by language regression and epileptic activity during sleep, presenting challenges in diagnosis and management due to its varied clinical manifestations. This case report of an 8-year-old girl with LKS contributes valuable insights into the clinical relevance, diagnostic criteria, and treatment outcomes of the syndrome. The successful use of valproic acid in improving language function highlights the importance of tailored pharmacological approaches and a multidisciplinary care approach. Atypical findings of sensorineural hearing loss and mild intellectual delay, in this case, expand the understanding of LKS presentations, emphasizing the heterogeneity of the syndrome. This case emphasizes the need for further research to enhance diagnostic accuracy, optimize therapeutic interventions, and improve long-term outcomes for individuals with LKS.

## Ethical approval

Our institution has exempted this study from ethical review.

## Consent

Written informed consent was obtained from the patient’s parents/legal guardian for publication and any accompanying images. A copy of the written consent is available for review by the Editor-in-Chief of this journal on request.

## Sources of funding

Not applicable.

## Author contribution

M.M.S., M.H.S., A.A.K., and T.W.A.: writing the manuscript; H.A.: reviewing and editing the manuscript.

## Conflicts of interest disclosure

All authors have no conflicts of interest to declare.

## Research registration unique identifying number (UIN)

Not applicable.

## Guarantor

Dr Hany Abueita.

## Data availability statement

Not applicable.

## Provenance and peer review

Not commissioned, externally peer-reviewed.
